# Mass Spectrometry-Based Metabolomics Revealed Effects of Metronidazole on *Giardia duodenalis*

**DOI:** 10.3390/ph16030408

**Published:** 2023-03-07

**Authors:** Supaluk Popruk, Amanee Abu, Sumate Ampawong, Tipparat Thiangtrongjit, Phornpimon Tipthara, Joel Tarning, Suthasinee Sreesai, Onrapak Reamtong

**Affiliations:** 1 Department of Protozoology, Faculty of Tropical Medicine, Mahidol University, Bangkok 10400, Thailand; 2 Department of Tropical Pathology, Faculty of Tropical Medicine, Mahidol University, Bangkok 10400, Thailand; 3 Department of Molecular Tropical Medicine and Genetics, Faculty of Tropical Medicine, Mahidol University, Bangkok 10400, Thailand; 4 Mahidol Oxford Tropical Medicine Research Unit, Faculty of Tropical Medicine, Mahidol University, Bangkok 10400, Thailand; 5 Centre for Tropical Medicine and Global Health, Nuffield Department of Clinical Medicine, University of Oxford, Oxford OX1 4BH, UK; 6 Central Equipment Unit, Faculty of Tropical Medicine, Mahidol University, Bangkok 10400, Thailand

**Keywords:** *Giardia duodenalis*, metabolomics, metronidazole, squamosinin A, glycerophospholipid metabolism

## Abstract

*Giardia duodenalis* is a significant protozoan that affects humans and animals. An estimated 280 million *G. duodenalis* diarrheal cases are recorded annually. Pharmacological therapy is crucial for controlling giardiasis. Metronidazole is the first-line therapy for treating giardiasis. Several metronidazole targets have been proposed. However, the downstream signaling pathways of these targets with respect to their antigiardial action are unclear. In addition, several giardiasis cases have demonstrated treatment failures and drug resistance. Therefore, the development of novel drugs is an urgent need. In this study, we performed a mass spectrometry-based metabolomics study to understand the systemic effects of metronidazole in *G. duodenalis*. A thorough analysis of metronidazole processes helps identify potential molecular pathways essential for parasite survival. The results demonstrated 350 altered metabolites after exposure to metronidazole. Squamosinin A and *N-*(2-hydroxyethyl)hexacosanamide were the most up-regulated and down-regulated metabolites, respectively. Proteasome and glycerophospholipid metabolisms demonstrated significant differential pathways. Comparing glycerophospholipid metabolisms of *G. duodenalis* and humans, the parasite glycerophosphodiester phosphodiesterase was distinct from humans. This protein is considered a potential drug target for treating giardiasis. This study improved our understanding of the effects of metronidazole and identified new potential therapeutic targets for future drug development.

## 1. Introduction

*Giardia duodenalis* (syn. *G*. *lamblia*, *G*. *intestinalis*) is an essential intestinal protozoan that causes giardiasis in humans and various animals [1,2,3,4]. The transmission route is mainly through the fecal–oral route by ingesting contaminated water or food and often exhibits zoonotic transmission [5,6,7]. Giardiasis is common worldwide in children and adults, especially in the elderly, travelers, and patients with weak immune systems [7,8,9]. Around 280 million *G*. *duodenalis* diarrheal cases are reported annually [2]. The prevalence of *Giardia* infection is higher in developing countries [7,10] due to poor sanitation and limited water treatment facilities [9,11]. In Thailand, the prevalence of giardiasis in humans is 0.4–37.7% due to the diverse populations, methods for detection, and locations [12]. The clinical manifestations of giardiasis range from asymptomatic to acute or chronic diarrheal cases [9]. The symptoms are more severe and persistent in children, the elderly, and immunocompromised patients [13,14,15]. There are no vaccination for giardiasis. Therefore, drug treatment is the only method to control this disease.

Drug therapies for treating giardiasis include 5-nitroimidazole derivatives (metronidazole, tinidazole, secnidazole, and ornidazole), benzimidazoles, nitazoxanide, furazolidone, quinacrine, chloroquine, and paromomycin [10,16]. Metronidazole is a 5-nitroimidazole first-line drug most commonly used for treating giardiasis. It was initially developed in the 1950s and approved by FDA in 1963. Currently, this drug constitutes the list of WHO essential medicines [17]. Metronidazole can act against *Trichomonas vaginalis*, *Entamoeba histolytica*, and anaerobic and microaerophilic bacteria [18]. Metronidazole is activated by reducing its nitro group under low oxygen concentrations and reacts with multiple targets in the cell [10,18,19]. In bacteria, 5-nitroimidazole drugs can cause DNA damage [20,21,22,23,24]. Structural proteins and redox proteins might involve in mechanisms of different nitro-heterocyclic compounds in *G*. *duodenalis* [25,26]. However, none of the global effects of metronidazole mechanisms has been reported, and it is unclear how the possible targets demonstrate an anti-giardial effect [22]. Despite the effectiveness of 5-nitroimidazole drugs, a significant number of giardiasis cases have shown treatment failures and drug resistance [27,28]. Drug development is an effective strategy to control this disease. In this study, we applied a mass spectrometry-based metabolomics analysis to understand the systemic effects of metronidazole in *G*. *duodenalis.* A half maximal inhibitory concentration (IC_50_) of metronidazole was evaluated on *G*. *duodenalis.* Transmission electron microscopy was used to reveal the morphological changes of *G*. *duodenalis* after metronidazole exposure. The metabolite profiling was performed using mass spectrometry to identify the differential metabolites effect by metronidazole. To identify the potential mechanisms of metronidazole, we carried out a pathway analysis. Understanding the mechanisms of metronidazole may help to identify potential routes that are essential for parasite survival. This strategy is an alternative to choosing potential new medication targets for treating giardiasis.

## 2. Results

### 2.1. Effects of Metronidazole on G. duodenalis

**Figure 1:**
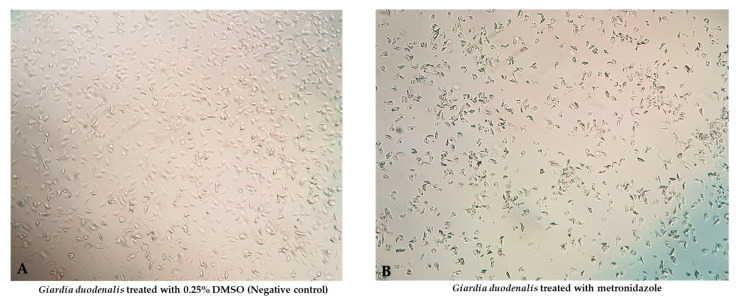
Morphological changes of *G. duodenalis* exposed to metronidazole: normal architecture of Giardia ((**A**); 0.25% DMSO (Negative control)) compared with degenerated Giardia ((**B**); metronidazole) were observed.

After 48 h of treatment, metronidazole effectively killed the parasites with a half maximal inhibitory concentration (IC_50_) of 2.013 ± 0.35 μg/mL (mean ± SD), and the inhibition were dose-dependent (Figure 1).

### 2.2. Conventional Transmission Electron Microscopy

**Figure 2:**
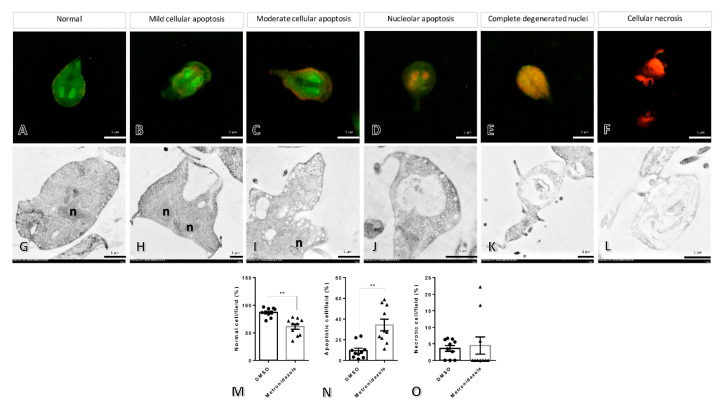
The morphological and ultrastructural changes in *Giardia* after treatment with metronidazole and 0.25% dimethyl sulfoxide. The different stages of the parasite were characterized by acridine orange/ethidium bromide staining (**A**–**F**), normal cells with whole green cytoplasm and nucleus (**A**), mild to moderate cellular apoptosis with diffused increasing orange in the cytoplasm ((**B**,**C**), respectively), nucleolar apoptosis to complete degenerative nuclei with orange nuclei until the entire cell is orange colored ((**D**,**E**), respectively), and necrosis with red cells (**F**). Ultrastructural changes in the parasite were demonstrated by electron microscopic study (**G**–**L**), intact cell (**G**), vacuolated degeneration ranging by low, moderate, and high severities and loss of normal cellular architecture (**H**–**K**), and complete loss of normal cellular components (**L**), the bar graphs comparing the number of normal, apoptosis, and necrosis cells in any treatment (**M**–**O**). n: nucleus, **: *p*-value < 0.01.

After the parasite was exposed to metronidazole, acridine orange/ethidium bromide (EB/AO), stain revealed that several stages of cellular degeneration were observed when compared to the 0.25% DMSO treated parasite (Figure 2A), including mild to moderate cellular apoptosis, nucleolar apoptosis, complete degenerative nuclei, and cellular necrosis (Figure 2B–F). Electron microscopic study characterized giardia ultrastructure relevant to the results obtained after EB/AO staining (Figure 2G–L). The semi-quantitative study indicated that the 0.25% DMSO-treated group had a significantly higher number of normal cells than the metronidazole-treated groups (Figure 2M). In addition, the apoptotic cells were significantly increased in the metronidazole group compared to the 0.25% DMSO-treated group (Figure 2N). However, there was no significant difference in necrotic cells between the control and metronidazole-treated groups (Figure 2O).

### 2.3. Metabolomics Analysis

**Figure 3:**
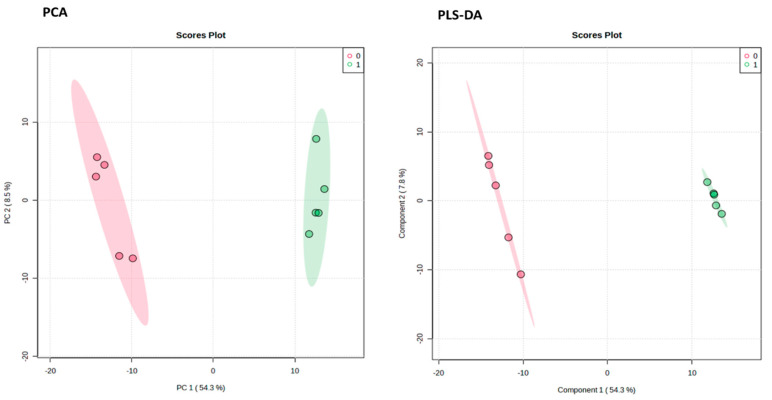
Pairwise analysis of metabolomic data using principal component analysis (**PCA**) and partial least squares–discriminant analysis (**PLS**-**DA**). Red and green represent the data from the control and metronidazole treatments, respectively.

The effects of metronidazole on adult *G. duodenalis* were examined using metabolomics analysis. The metabolite profiles of *G. duodenalis* trophozoites treated with metronidazole at a concentration equivalent to IC_50_ and 0.25% dimethyl sulfoxide (DMSO) (control) were examined. The overlaid total ion chromatograms of QC samples were shown in Figure S1. For data analysis, principal component analysis (PCA) and partial least squares–discriminant analysis (PLS-DA) were introduced (Figure 3).

**Figure 4:**
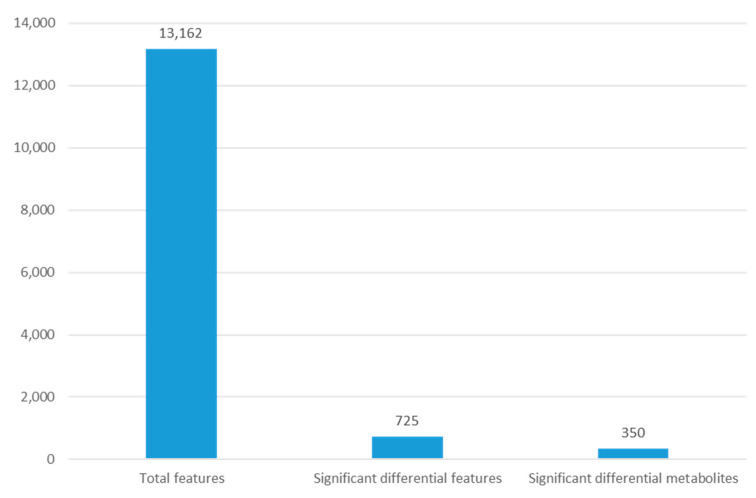
The number of features observed for metabolomic analysis of *G. duodenalis* after metronidazole exposure.

The outcomes demonstrated that the *G. duodenalis* metabolite profile following treatment with metronidazole differed from that of the control. The mass spectrometric analysis allowed for the observation of 13,162 distinct features. After exposure to metronidazole, *G. duodenalis* showed differences in 725 features. The METLIN database identified 350 of these metabolites (Figure 4).

**Figure 5:**
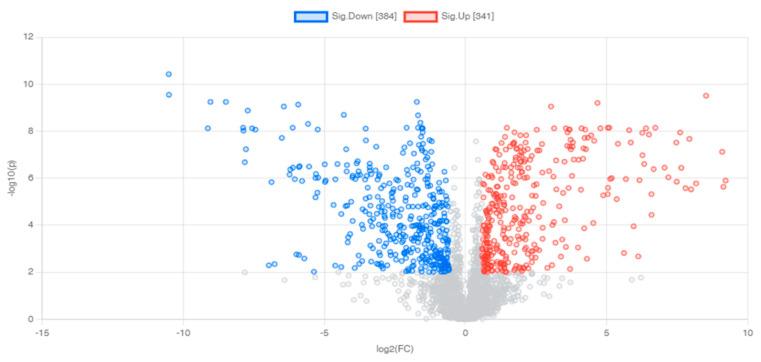
Volcano plots demonstrating differential metabolites of *G. duodenalis* after metronidazole treatment. Horizontal lines represent *p*-values equal to 0.01. Vertical lines represent fold changes equal to 1.5 and −1.5, respectively. Blue and red refer to down- and up-regulated metabolites, respectively.

A volcano plot was created to display statistical significance (*p*-value) vs. the degree of change (fold change), as shown in Figure 5. After exposure to metronidazole, 725 differential features were used for *p*-value 0.01 and folded change 1.5. Around 341 features were up-regulated, and 384 were down-regulated. Following metabolite identification using the METLIN database, there were 193 up- and 211 down-regulated metabolites (Table 1, Table 2 and Table S1).

**Figure 6:**
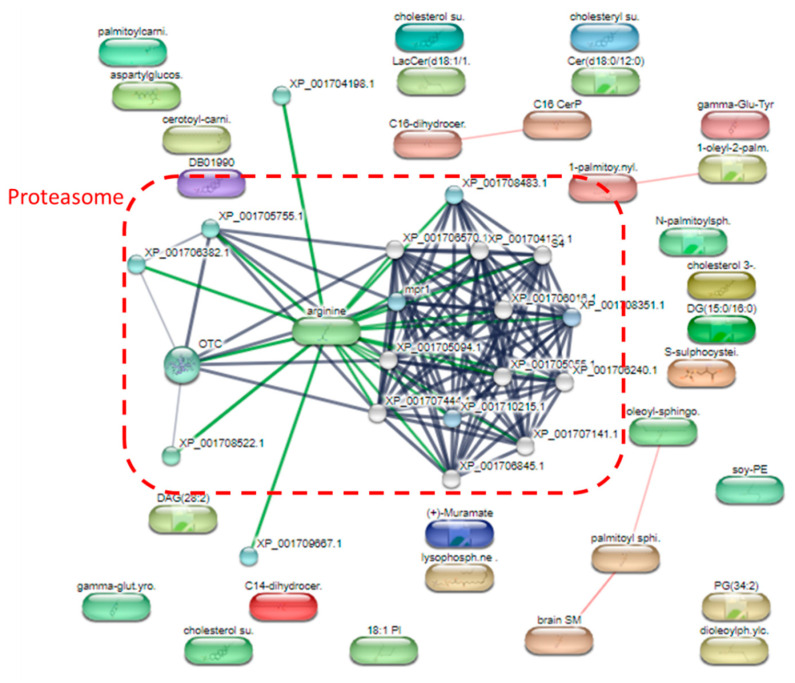
A detailed analysis of the pathways of significantly altered metabolites after metronidazole exposure.

The most up-regulated metabolites following treatment with metronidazole were squamosinin A, octacosanal, and acarbose, as measured by the fold change. In addition, several sphingolipids were also up-regulated after metronidazole exposure; for example, Cer(d18:0/12:0), GlcCer(d16:1/23:0), Cer(d18:0/12:0), LacCer(d18:0/22:0), and Cer(d18:0/14:0). The most down-regulated metabolites after treatment with metronidazole were *N*-(2-hydroxyethyl)hexacosanamide, C-8 ceramine, and amotidine sulfoxide. Phopholipids were also down-regulated after metronidazole exposure such as PG(12:0/21:0) and PC(22:6(4Z,7Z,10Z,13Z,16Z,19Z)/22:6(4Z,7Z,10Z,13Z,16Z,19Z))[S]. The STITCH bioinformatics tool performed pathway analysis on the different metabolites (Figure 6). Proteasome metabolism was a crucial pathway for differential metabolites.

**Figure 7:**
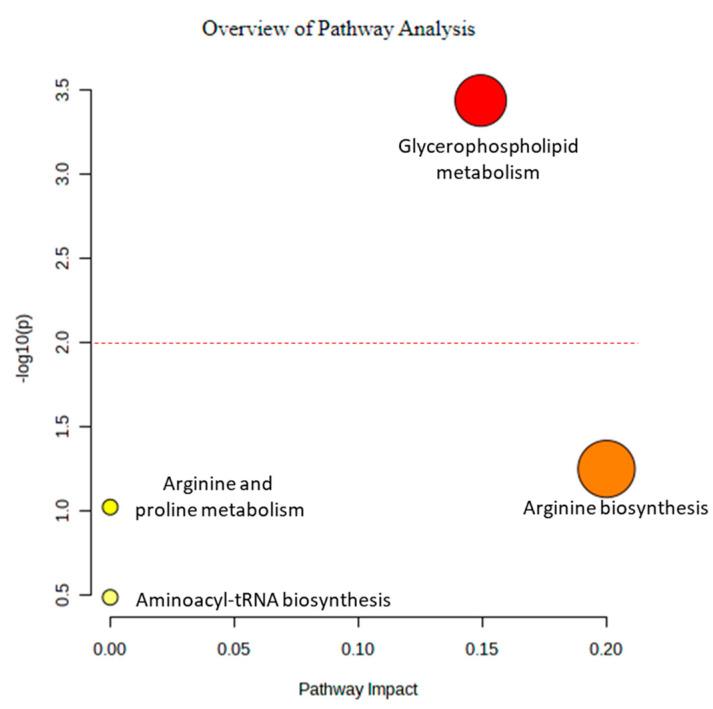
Metabolite annotation and pathway analyses using MetaboAnalyst.

Further detailed analysis of the pathways of the altered metabolites was performed using MetaboAnalyst (Figure 7). The findings indicated that glycerophospholipid metabolism was a prominent mechanism involved with the altered metabolites. Therefore, glycerophospholipid metabolism might be a potential pathway for drug development.

**Figure 8:**
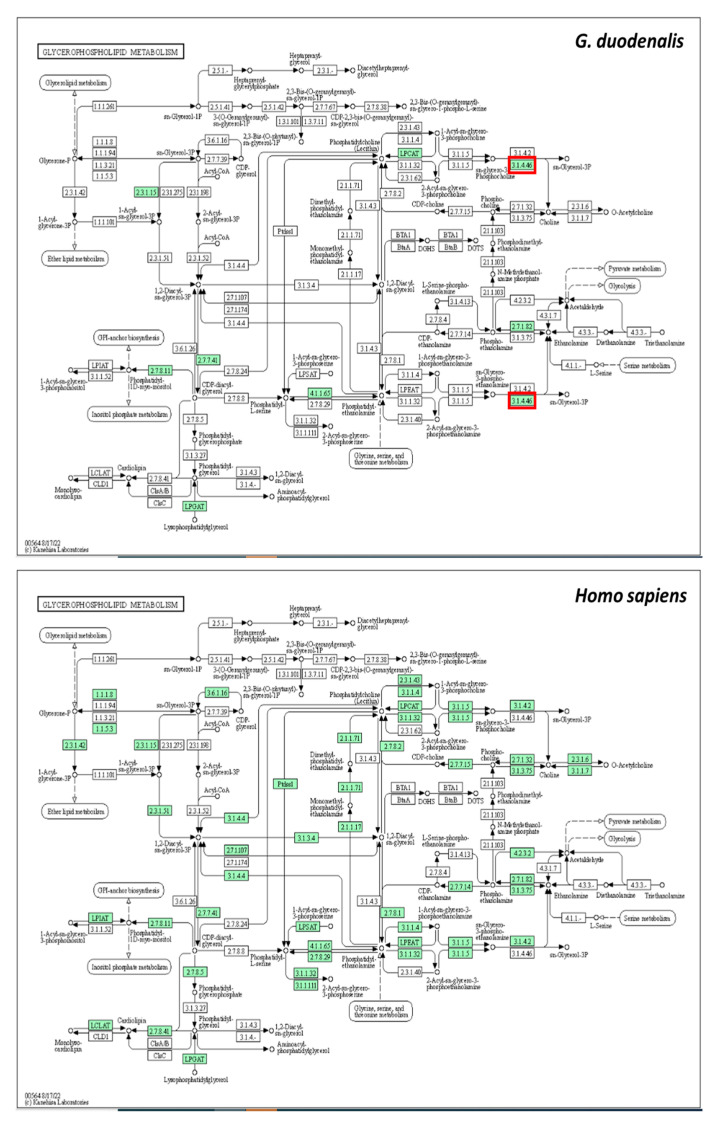
Glycerophospholipid metabolism of *G. duodenalis* and *Homo sapiens*. Green boxes represent proteins that play a role in each organism. Red squares represent proteins that play a role only in *G. duodenalis*.

Since glycerophospholipid metabolism was the most significant pathway affected by metronidazole, this pathway from *G. duodenalis* and *Homo sapiens* was compared using the Kyoto Encyclopedia of Genes and Genomes pathway database (Figure 8). According to the results obtained, glycerophosphodiester phosphodiesterase (EC:3.1.4.46), shown in red square in Figure 7, plays a role only in *G. duodenalis* glycerophospholipid metabolism. In contrast, glycerophosphocholine phosphodiesterase GPCPD1 (EC:3.1.4.2) plays a role in *H. sapiens* glycerophospholipid metabolism. The sequence similarity between *G. duodenalis* and *H. sapiens* is only 24.38%. *G. duodenalis* glycerophosphodiester phosphodiesterase might be a potential drug target for treating giardiasis.

## 3. Discussion

This study demonstrated in vitro antiparasitic activity of metronidazole with an IC_50_ value of 2.013 ± 0.35 μg/mL (11.67 µM). The IC_50_ value was in the same range as reported in another in vitro study with an IC_50_ value of 2.5 µM for metronidazole [29]. This outcome demonstrated the reliability of our antiparasitic test. According to metabolomic analysis, the most up-regulated metabolite following metronidazole treatment was squamosinin A. Squamosinin A is a member of organic annonaceous acetogenins. They are waxy fatty acid derivatives (often C32 or C34), which combine a terminal carboxylic acid with a 2-propanol unit at the C-2 position to create an alpha, beta, unsaturated, and gamma-lactone that has been replaced with a methyl group. However, there has been little information on this metabolite. It is presumed to function as a human membrane stabilizer [30]. Membrane stabilization plays a role in local anesthetics [31]. In parasites, the up-regulated squamosinin A may lead to immobility of *G. duodenalis* and could be further eliminated by host immunity. Acarbose was up-regulated in *G. duodenalis* after treatment with metronidazole. This metabolite has been used for treating diabetics as a specific inhibitor of glucosidase-like proteins. In addition, it demonstrated in vitro and in vivo antileishmanial activity against *Leishmania infantum* [32]. The up-regulated acarbose may have antiparasitic effects in *G. duodenalis*, similar to the *Leishmania* parasite. 3-Demethylubiquinone-9 increased in *G. duodenalis* after treatment with metronidazole. It belongs to the class of organic ubiquinones, which are coenzyme Q derivatives. Ubiquinone is crucial for regulating oxidative stress and electron transport pathways associated with membrane energization in *Giardia* [33]. The up-regulation of this metabolite might control oxidative stress following drug exposure. The most down-regulated metabolites after exposure to metronidazole were *N*-(2-hydroxyethyl)hexacosanamide. This metabolite is found in the human peripheral tissues and is proposed to have anti-inflammatory effects [34]. Cell death plays an essential role in the regulation of inflammation. According to our electron microscopy experiments, apoptotic and necrosis cells were significantly increased in the metronidazole-treated group. The down-regulation of anti-inflammatory molecules might relate to cell death observed after metronidazole treatment. Lignoceric acid decreased after treatment with metronidazole. It is a saturated fatty acid in the *Trypanosoma cruzi* epimastigote structure [35]. The down-regulation of structural metabolite might be involved in the damage of ultrastructure after treatment with metronidazole, as observed by electron microscopy. Hexacosanoyl carnitine, an acylcarnitine, was down-regulated after metronidazole exposure. The deficiency of down-regulation in the production and excretion of unusual acylcarnitines could lead to several disorders. In humans, an uncommon condition called acylcarnitine deficiency prevents long-chain fatty acids from being used for mitochondrial beta-oxidation and ketogenesis. It might manifest as a severe clinical form during the neonatal period or infancy, usually accompanied by convulsions, hypothermia, encephalopathy, cardiomyopathy, and liver failure, or as a milder phenotype with bouts of hypoglycemia and hyperammonaemia during concurrent illness [36]. The aberrant function in *G. duodenalis*, which is comparable to humans, may be due to the down-regulation of hexacosanoyl carnitine.

Due to the pathway analysis by the STITCH bioinformatics tool, proteasome metabolism was a crucial pathway for differential metabolites. Both pathogenic circumstances and cell physiology benefit from regulated proteolysis. In most cases, regulated proteolysis is carried out by the ubiquitin- and proteasome-dependent proteolytic system, which is also in charge of the bulk of cytoplasmic proteolysis [37]. In our electron microscopic experiments, the majority of cytoplasmic proteolysis was observed. This finding relates to the metabolomics data that the proteasome-dependent proteolytic system regulates apoptosis.

Additionally, the known mechanisms of metronidazole are that it enters the body through diffusion, interferes with DNA to prevent protein synthesis, damages DNA strands, and impairs helical DNA structure. Therefore, it results in cell death [38]. Proteasome assembly and functions are induced by suppressing protein synthesis [39]. The observation of proteasome as a significant pathway in the *G. duodenalis* in our study may be the consequence of protein synthesis inhibition of metronidazole.

According to the pathway analysis by MetaboAnalyst, an essential pathway for the altered metabolites was glycerophospholipid metabolism. The parasite membrane’s primary components are glycerophospholipids, primarily produced by the enzymatic machinery encoded by the parasite [40]. Glycerophospholipids are hydrophilic head groups linked via phosphate to glycerol-bound fatty acid or fatty alcohol chains. In *Plasmodium*, intraerythrocytic glycerophospholipids’ metabolism has identified possible targets for chemotherapeutic interventions [41]. Metronidazole could disturb the order and packing of phospholipids in the cell membrane [42]. According to our results, metronidazole might affect the glycerophospholipids leading to interference in the parasite membrane. Comparing the glycerophospholipid metabolisms of *G. duodenalis* and humans, the parasite glycerophosphodiester phosphodiesterase was distinct from humans. This protein is considered a potential drug target for giardiasis treatment. In trypanosomes, phosphodiesterases are essential for infectivity and survival [43]. Phosphodiesterase inhibitor has been studied as a new-generation antiprotozoal medication [44]. Pyrazolones are heterocyclic compounds that could inhibit phosphodiesterases. This inhibitor demonstrated anti-*Trypanosoma cruzi* activity in experimental mouse models [45]. Like *T. cruzi*, phosphodiesterase inhibitors may be a novel therapeutic alternative for giardiasis.

## 4. Materials and Methods

### 4.1. G. duodenalis Culture Conditions

Trophozoites of the in-house *G. duodenalis* strain were cultured anaerobically in a modified TYI-S-33 medium (Trypticase–yeast extract–iron–serum medium) [46]. The medium was supplemented with 14% heat-inactivated bovine serum and 3% NCTC-135. The cell growth and viability of trophozoites were examined after incubation for 24 h using an inverse microscope. The log-phase cultures (2–3 days) were harvested on ice for 20 min and centrifuged at 1500 rpm for 15 min at 4 °C. The trophozoites were counted in a hemocytometer and used for the study.

### 4.2. In Vitro Anti-giardia Assay

Metronidazole (Sigma-Aldrich, St Louis, MO, USA) was dissolved in 100% dimethyl sulfoxide (DMSO) and was serially diluted, ranging from 0.01 to 10µg/mL. The culture medium was used as blank, and 0.25% DMSO was used as a negative control; this concentration did not affect the trophozoites. Briefly, different concentrations of metronidazole, negative control, and blank were added to white opaque-walled 96-well microplates (Perkin Elmer, Waltham, MA, USA). Then, 2 × 10^5^ trophozoites were added to each well, except blank wells, to make a final volume of 100 µL. The final concentration of the DMSO was 0.25%, which did not affect the test. All experiments were performed in triplicate. The plates were sealed and incubated at 37 °C for 48 h under anaerobic conditions in 2.5-L Pack-Rectangular Jars (*Mitsubishi* Gas Chemical Co., Inc., Tokyo, Japan). The tested microplates were incubated for 48 h, and subsequent 100 µL BacTiter-Glo^TM^ Microbial Cell Viability Assay fluid was added directly to each well. The tested microplates were mixed on an orbital shaker and incubated for 20 min at 37 °C before trophozoite viability was recorded using luminescence.

The percentage trophozoite viability at various concentrations of the metronidazole was determined using the following formula:

% cell survival = ((sample luminescence − culture medium luminescence)/(non-treated control luminescence − culture medium luminescence)) × 100

% inhibition = 100 − % trophozoites that survived

The inhibitory concentration at which 50% of parasites were killed (IC_50_) was defined as the concentration of metronidazole required to inhibit the growth of *Giardia* trophozoites by 50%.

### 4.3. Ethidium Bromide/Acridine Orange (EB/AO) Staining

The apoptotic cells induced by metronidazole were identified using EB/AO dual staining [47]. Two microliters of the mixture of 100 µg/mL EB and AO dyes were added to 50 µL of giardia suspension. Cellular morphology and apoptotic number were examined under the fluorescent microscope in at least ten areas of the high-power field (400×)/group. Different stained colors represent different stages of cell architecture: green; normal cells, orange; apoptotic cells, and red; necrotic cells.

### 4.4. Conventional Transmission Electron Microscopy

The giardia pellets were fixed in each group with 2.5% glutaraldehyde and 1% osmium tetroxide for 1 h at room temperature, respectively. The fixed pellets were dehydrated with a graded ethanol series, infiltrated, embedded in LR white resin (EMS, Sumter, SC, USA), polymerized in a 65 °C oven for 24–48 h, and cut into 100 nm thickness. The sections were examined under a transmission electron microscope (Hitachi; model HT7700, Tokyo, Japan) to identify parasitic ultrastructural changes during treatment [12].

### 4.5. Metabolite Extraction

The metabolites were extracted following a previously described protocol [48]. *G. duodenalis* trophozoites treated with metronidazole at an IC_50_ concentration (2.01 µg/mL) and 0.25% DMSO (control) were homogenized in 500 μL methanol, snap-frozen in liquid nitrogen, and thawed. The supernatant was collected by centrifugation at 800× *g* for 1 min at 4 °C. The pellet was subjected to the same protocol. The supernatant from the first and second extraction was pooled. The pellet was resuspended in 250 μL H_2_O, snap-frozen in liquid nitrogen, and thawed. The supernatant was collected by centrifugation at 15,000× *g* for 1 min at 4 °C and pooled into the methanol extract. The pooled extract was centrifuged at 15,000× *g* for 1 min at 4 °C, and the remaining debris was discarded. The clear supernatant was dried with a speed vacuum (Tomy Digital Biology, Tokyo, Japan). Five biological replicates and two technical replicates were performed for metabolomics analysis.

### 4.6. Mass Spectrometric Analysis

Ultra-high performance liquid chromatography (Agilent 1260 Quaternary pump, Agilent 1260 High-Performance Autosampler and Agilent 1290 Thermostatted Column Compartment SL, Agilent Technologies, Santa Clara, CA, USA) coupled with DuoSpray ion source electrospray ionization (ESI) quadrupole time-of-flight mass spectrometer (Q-TOF-MS) (TripleTOF 5600+, SCIEX, Framingham, MA, USA) was used for analysis. Mobile phases A and B were 0.1% formic acid in water and 0.1% formic acid in acetonitrile, respectively. Mobile phases A and B were mixed 1:1 (*v*/*v*) to dissolve the dried metabolite samples. After being transferred to an LC vial, the solution was stored in the auto-sampler at 6 °C until analysis. A solution volume of five microliters was injected into the UHPLC. The UPLC separation was performed by a C18 reversed-phase column (ACQUITY UPLC BEH, 2.1 × 100 mm, 1.7 µM, Waters). The flow rate was set at 0.3 mL/min, and the column oven was 40 °C. Gradient elution began at 5% B and continued for 2 min (0.0–2.0 min). In 0.5 min (2.0–2.5 min), the gradient ramped to 60% B, and in 1.5 min, it reached 80% B. (2.5–4.0 min). In 8 min (4.0–12.0 min), the gradient rose to 100% B, then remained steady for 5 min (12.0–17.0 min). With a 2.9 min re-equilibration period (17.1–20.0 min) before the next injection, the gradient reached 5% B in 0.1 min (17.0–17.1 min). Analyst Software version 1.7 (SCIEX) was used to acquire the data. Metabolomic analyses were performed in positive (+ESI) and negative (-ESI) electrospray ionization modes. The mass range of the TOF-MS scans was *m*/*z* 100–1000, and the MS/MS ion scans were *m*/*z* 50–1000. The quality control (QC) samples were created by pooling identical aliquots of each metabolite sample. To evaluate the system’s effectiveness, we injected the QC samples before, during, and after sample analysis.

### 4.7. Data Analysis

The XCMS online software version 3.7.1 (https://xcmsonline.scripps.edu/landing_page.php?pgcontent=mainPage) was used to analyze the raw mass spectra files in the wiff and.wiff.scan file formats (The Scripps Research Institute, La Jolla, CA, USA). The “Pairwise” mode with the “UPLC/Triple TOF pos” technique was used to compare the control and treated groups. Feature extraction, alignment, annotation, and identification were all criteria for metabolite annotation. Positive and negative polarity were selected for each set of data in order to extract features. The maximum permitted *m*/*z* deviation was set at 15 ppm. The signal/noise threshold was 6, and the minimal m/z difference was 0.01. For alignment, the maximum fraction allowed was 0.5, the minimum retention time duration was 5, and the minimum overlapped *m*/*z* width was 0.015. The error for annotation was 5 ppm, the *m*/*z* absolute error was 0.01, and isotopic characteristics and their adduct forms were searched for. In order to identify them, 74 common adducts were taken into account for a database search with a tolerance of 5 ppm. The metabolite annotation procedure was performed using the METLIN database. The MetaboAnalyst online program version 5.0 (https://www.metaboanalyst.ca; pang et al., 2021) was used to evaluate the metabolomic data from the XCMS under the “Statistical Analysis (one factor)” and “Pathway Analysis” modules. Quantile normalization, cube root data transformation, and data range scaling were used to normalize the metabolites and their concentrations for the statistical analysis module. PCA, partial least squares–discriminant analysis (PLS-DA), and volcano plot were used for data visualization. The 95% confidence areas for PCA and PLS-DA were shown. To create the volcano graphic, log2 of fold change and −log of *p*-value were employed. Different metabolites were found when using the specific criterion (>1.5 fold change, *p*-value 0.01). The STITCH database version 5.0 (http://stitch.embl.de; Szklarczyk et al., 2016) and MetaboAnalyst were used for pathway analyses of differential metabolites, with a *p*-value of less than 0.01, indicating statistical significance.

### 4.8. Sequence alignment

Using the Blastp software, sequence alignment and identity calculations were carried out. The non-redundant protein sequence database of the NCBI was used to retrieve sequences. XP_001709226.1 and NP_057725.1 were *G. duodenalis* and *H. sapiens* glycerophosphodiester phosphodiesterase, respectively.

## 5. Conclusions

This research provided a better understanding of the effects of metronidazole via *G. duodenalis* proteasome and glycerophospholipid metabolisms. In addition, *G. duodenalis* glycerophosphodiester phosphodiesterase is a potential drug target for treating giardiasis. Identification of glycerophosphodiester phosphodiesterase inhibitors might be a novel potential drug for anti-giardia therapy.

## Figures and Tables

**Table 1:** Top-twenty up-regulated metabolites of *G. duodenalis* after treatment with metronidazole.

No.	Potential Metabolites	*m*/*z*	Mass Error (ppm)	log2(FC)	*p*-Value
1	Squamosinin A	621.4373	0	7.4866	1.43 × 10^−6^
2	octacosanal	426.467	0	7.4709	3.09 × 10^−8^
3	Acarbose	644.2434	4	7.054	3.59 × 10^−7^
4	PGPC	590.3465	1	6.3959	7.61 × 10^−9^
5	3-Demethylubiquinone-9	513.5355	2	6.2659	1.18 × 10^−8^
6	Cer(d18:0/12:0)	484.4725	0	6.1207	0.002144
7	GlcCer(d16:1/23:0)	814.616	2	5.9571	0.000112
8	Cer(d18:0/12:0)	501.4987	1	5.6833	1.10 × 10^−6^
9	LacCer(d18:0/22:0)	765.6695	0	5.616	0.00154
10	Cer(d18:0/14:0)	529.53	1	5.4023	3.46 × 10^−8^
11	C8-Dihydroceramide	456.4408	1	5.1804	0.000001
12	PC(22:6(4Z,7Z,10Z,13Z,16Z,19Z)/22:6(4Z,7Z,10Z,13Z,16Z,19Z))[S]	558.4361	0	5.129	1.09 × 10^−6^
13	DL-Cerebronic acid	402.3941	0	5.0947	7.21 × 10^−9^
14	2-amino-14,16-dimethyloctadecan-3-ol	398.4362	0	5.0425	7.21 × 10^−9^
15	*N*-(2-hydroxyethyl)hexacosanamide	485.5042	0	5.0409	4.81 × 10^−6^
16	tetracosanal	370.4047	1	4.5593	2.57 × 10^−8^
17	Glycerol 2-(9Z,12Z-octadecadienoate) 1-hexadecanoate 3-O-[alpha-D-galactopyranosyl-(1–>6)-beta-D-galactopyranoside]	915.601	4	4.3026	0.002712
18	L-Arginine	175.119	0	4.2669	5.92 × 10^−8^
19	22-methyl-tricosanoic acid	386.3993	0	4.2412	8.68 × 10^−9^
20	PS(21:0/0:0)	566.3447	3	4.206	0.000137

**Table 2:** Top-twenty down-regulated metabolites of *G. duodenalis* after treatment with metronidazole.

No.	Potential Metabolites	*m*/*z*	Mass Error (ppm)	log2(FC)	*p*-Value
1	*N*-(2-hydroxyethyl)hexacosanamide	485.5042	0	−10.517	3.79 × 10^−11^
2	C-8 Ceramine	457.4729	0	−10.511	2.86 × 10^−10^
3	Famotidine sulfoxide	336.0372	0	−7.5605	7.61 × 10^−9^
4	SM(d18:1/16:0)	703.5747	0	−6.876	1.49 × 10^−6^
5	9Z-Pentatriaconte	513.5355	2	−6.7659	4.56 × 10^−3^
6	WIN56291	338.0346	0	−6.5084	1.95 × 10^−8^
7	SM(d18:1/16:0)	703.5745	0	−6.2087	4.31 × 10^−7^
8	SM(d18:1/16:0)	725.5566	0	−6.0514	1.11 × 10^−6^
9	C6 CERAMIDE	430.3895	0	−5.9306	7.45 × 10^−10^
10	N4-Phosphoagmatine	955.7572	1	−5.8778	3.11 × 10^−7^
11	23-Hexacosen-1-ol	398.436	1	−5.5802	4.95 × 10^−9^
12	SM(d18:1/18:1(9Z))	729.5898	1	−5.3147	9.88 × 10^−7^
13	SM(d18:1/16:0)	725.5568	0	−4.9543	1.26 × 10^−6^
14	Lignoceric acid	386.3992	0	−4.538	2.52 × 10^−7^
15	CETRIMONIUM	312.3629	0	−4.3126	2.04 × 10^−9^
16	PG(12:0/21:0)	759.5147	0	−4.2497	1.55 × 10^−5^
17	Hexacosanoyl carnitine	523.472	0	−4.2286	5.92 × 10^−8^
18	Cer(d18:0/14:0)	529.5302	0	−4.2116	5.21 × 10^−7^
19	PC(22:6(4Z,7Z,10Z,13Z,16Z,19Z)/22:6(4Z,7Z,10Z,13Z,16Z,19Z))[S]	558.4364	1	−4.2024	2.79 × 10^−7^
20	SM(d18:1/18:1(9Z))	729.5908	0	−4.1938	1.48 × 10^−5^

## Data Availability

Not applicable.

## Supplementary Materials

The following supporting information can be downloaded at: https://www.mdpi.com/article/10.3390/ph16030408/s1, Table S1: Differential *G. duodenalis* metabolites after metronidazole exposure. Figure S1: Total ion chromatogram showing QC samples overlaid.
