# Supplementary material for: Mass Spectrometry-Based Metabolomics Revealed Effects of Metronidazole on Giardia duodenalis
Source: Pharmaceuticals (Basel). 2023 Mar 7;16(3):408. doi: 10.3390/ph16030408 (PMC10052756; doi:10.3390/ph16030408)
Supplement: Supplementary file 1 [file pharmaceuticals-16-00408-s001.zip › Figure S1.pdf]

Positive mode

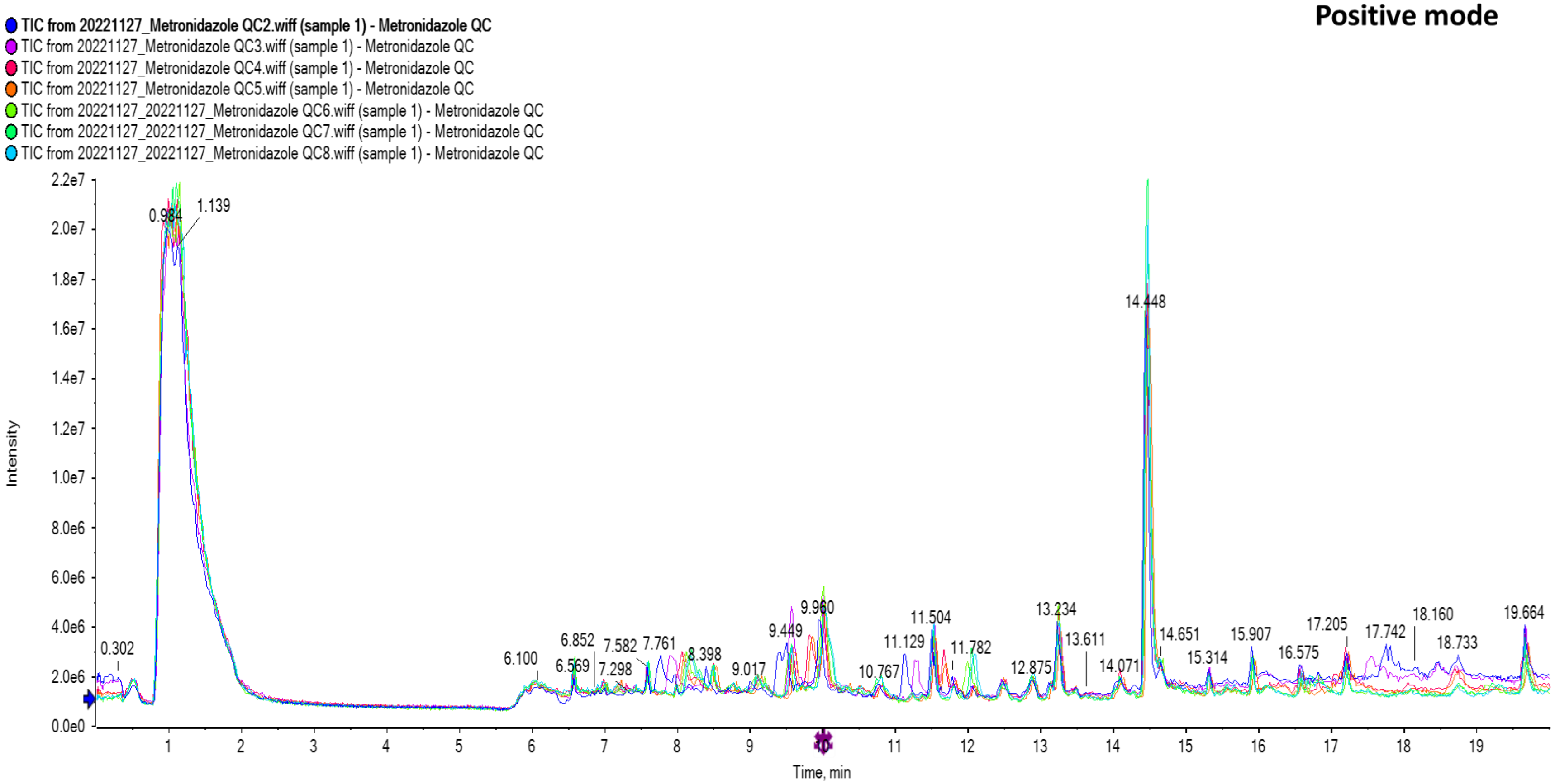

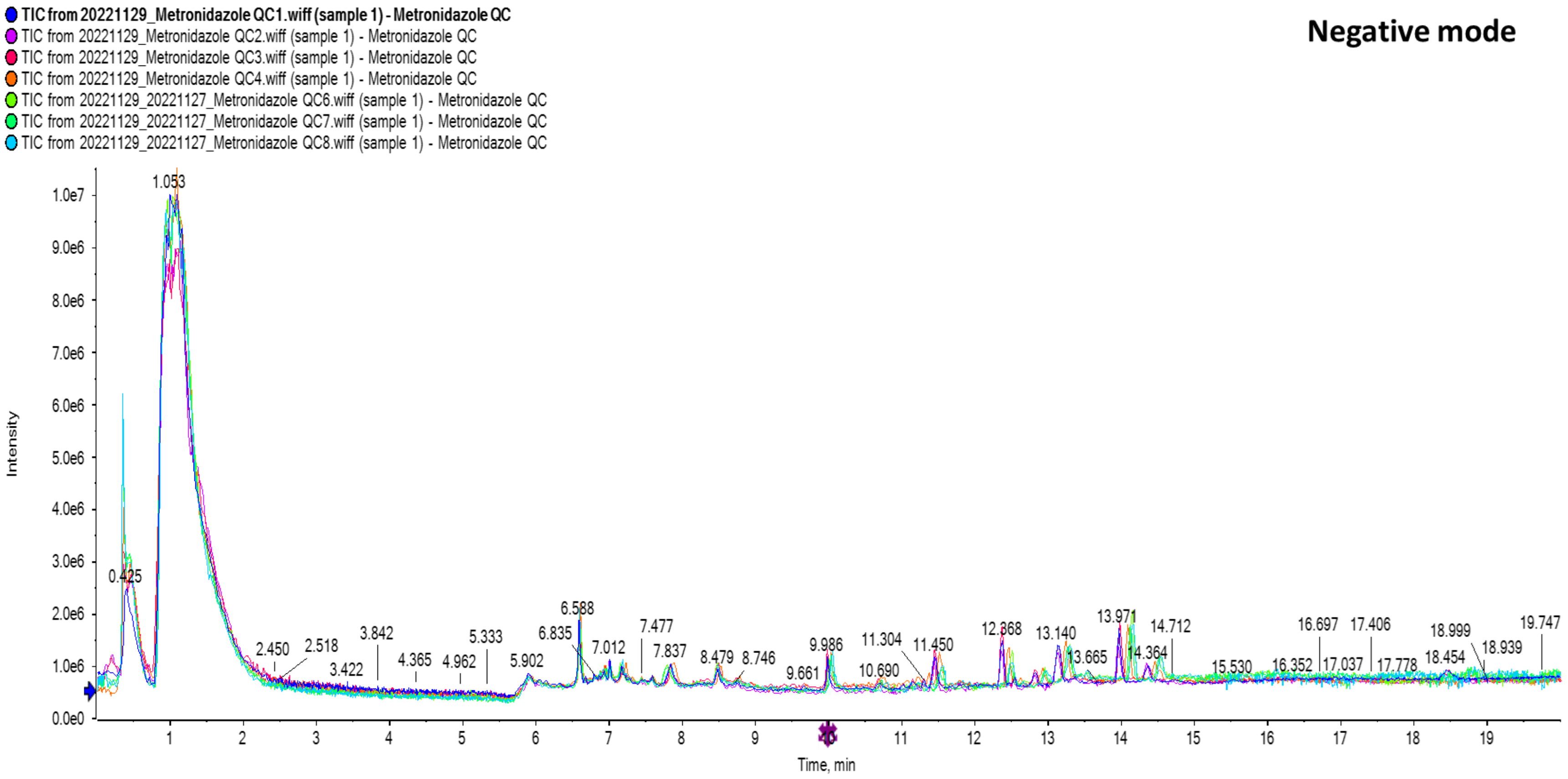

Figure S1. Total ion chromatogram showing QC samples overlaid. The methodological validation using quality control (QC) were performed. Aliquots (50 µL) from each of extract samples were mixed as QC samples. Fourteen QC samples were injected before sample analysis to condition the column. Aliquots of the same pooled QC were also injected between and after the samples for monitoring analytical variation. Overlaid QC sample chromatogram is shown in Figure S1.
